# Supplementary material for: Time-resolved phenotyping at subcellular resolution reveals shared principles and key trade-offs across antimicrobial peptide activities
Source: bioRxiv. 2026 Jul 24:2025.04.10.648262. Preprint. [Version 4] doi: 10.1101/2025.04.10.648262 (PMC13420454; doi:10.1101/2025.04.10.648262)
Supplement: Supplementary file 1 [file NIHPP2025.04.10.648262v4-supplement-2.pdf]

bioRxiv preprint doi: https://doi.org/10.1101/2025.04.10.648262; this version posted July 24, 2026. The copyright holder for this preprint (which was not certified by peer review) is the author/funder, who has granted bioRxiv a license to display the preprint in perpetuity. It is made available under a CC-BY 4.0 International license.

## Figure S1

**A**

| Name (abbreviation) | Molecular weight (kDa) | Net charge | Lysine fraction | Arginine fraction | Gravy index | Proline fraction | Hydrophobic fraction | Polar fraction |
|---|---|---|---|---|---|---|---|---|
| Bactenecin-7 (Bac7) | 4.21 | 11 | 0.0 | 0.31 | -1.41 | 0.46 | 0.2 | 0.8 |
| CRAMP | 3.88 | 6 | 0.24 | 0.03 | -0.89 | 0.06 | 0.29 | 0.71 |
| Camel Bactenecin (CamBac) | 4.98 | 13 | 0.0 | 0.32 | -1.11 | 0.37 | 0.24 | 0.76 |
| Cecropin A (CecA) | 4.0 | 7 | 0.19 | 0.03 | -0.07 | 0.03 | 0.46 | 0.54 |
| DJK-5 | 1.55 | 5 | 0.0 | 0.33 | 0.08 | 0.0 | 0.58 | 0.42 |
| Human beta-defensin 3 (HBD-3) | 5.16 | 11 | 0.13 | 0.16 | -0.7 | 0.02 | 0.33 | 0.67 |
| Indolicidin (Indo) | 1.91 | 4 | 0.08 | 0.15 | -1.07 | 0.23 | 0.54 | 0.46 |
| LL-37 | 4.49 | 6 | 0.16 | 0.14 | -0.72 | 0.03 | 0.35 | 0.65 |
| Magainin-2 (Mag2) | 2.47 | 3 | 0.17 | 0.0 | 0.08 | 0.0 | 0.43 | 0.57 |
| Melittin (Mltt) | 2.85 | 6 | 0.12 | 0.08 | 0.27 | 0.04 | 0.46 | 0.54 |
| PR-39 | 4.72 | 11 | 0.0 | 0.26 | -1.31 | 0.49 | 0.21 | 0.79 |
| Protegrin-1 (PG-1) | 2.16 | 7 | 0.0 | 0.33 | -0.25 | 0.0 | 0.44 | 0.56 |
| Tachyplesin-1 (Tac1) | 2.26 | 7 | 0.06 | 0.29 | -0.52 | 0.0 | 0.47 | 0.53 |
| Tur1A | 3.97 | 10 | 0.0 | 0.31 | -1.12 | 0.38 | 0.25 | 0.75 |
| Tur1B | 4.03 | 6 | 0.03 | 0.19 | -1.0 | 0.31 | 0.34 | 0.66 |

**B**

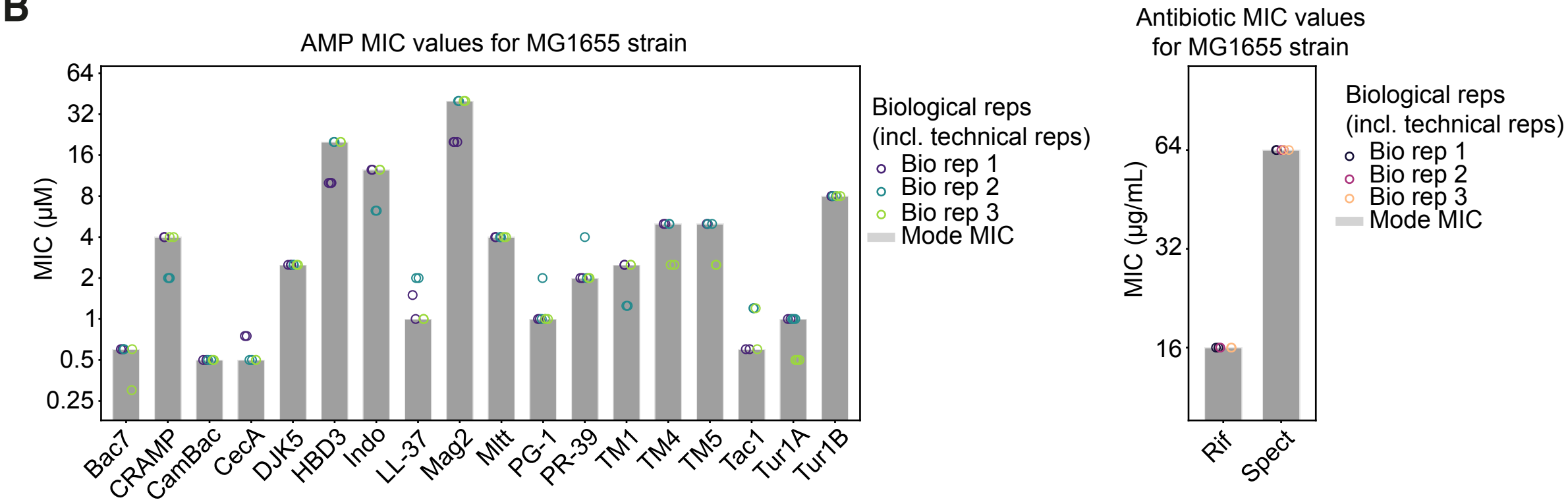


**C**

| AMP | Mode MIC | Unit |
|---|---|---|
| Bac7 | 0.6 | µM |
| CRAMP | 4 | µM |
| CamBac | 0.5 | µM |
| CecA | 0.5 | µM |
| DJK5 | 2.5 | µM |
| HBD3 | 20 | µM |
| Indo | 12.5 | µM |
| LL-37 | 1 | µM |
| Mag2 | 40 | µM |
| Melittin | 4 | µM |
| PG-1 | 1 | µM |
| PR-39 | 2 | µM |
| TM1 | 2.5 | µM |
| TM4 | 5 | µM |
| TM5 | 5 | µM |
| Tac1 | 0.6 | µM |
| Tur1A | 1 | µM |
| Tur1B | 8 | µM |
| Rif | 16 | µg/mL |
| Spect | 64 | µg/mL |

**D**

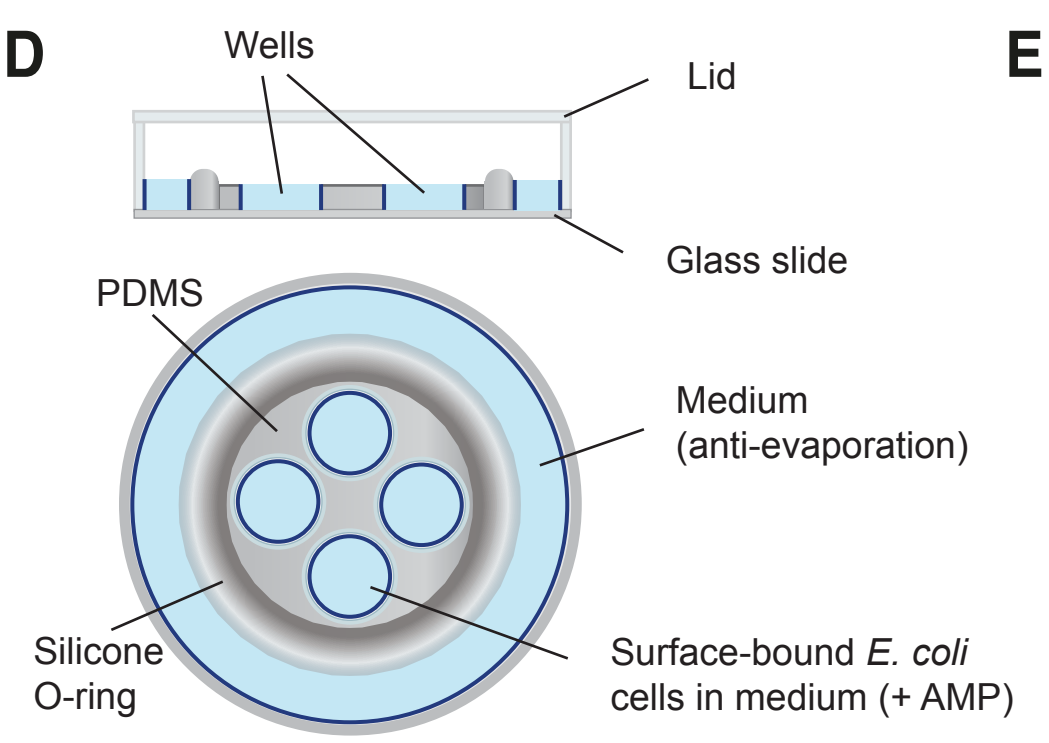


**E**

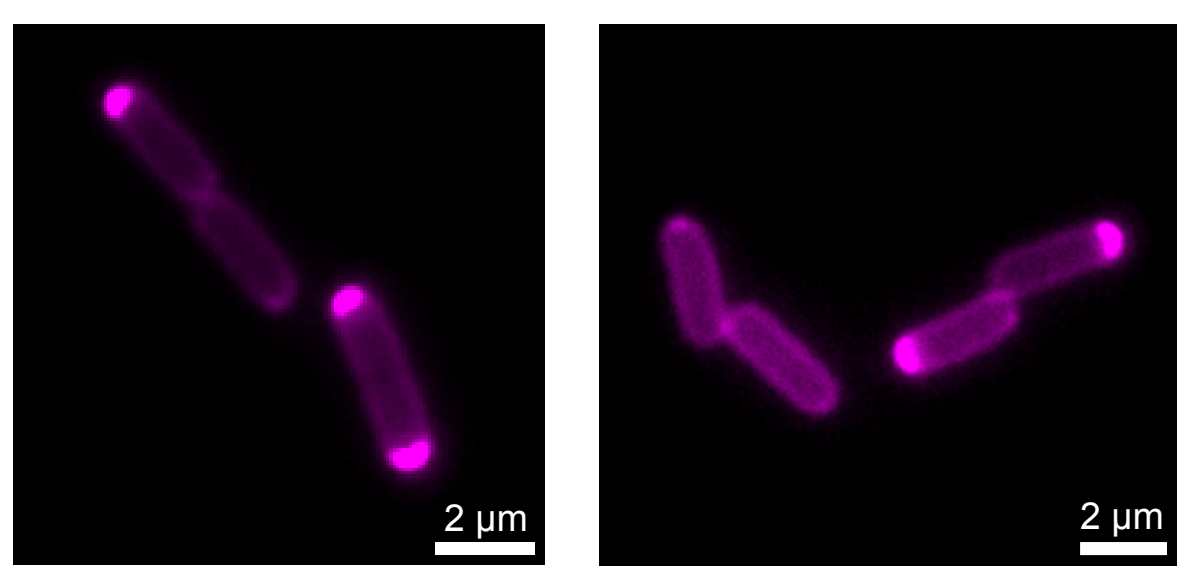


**F**

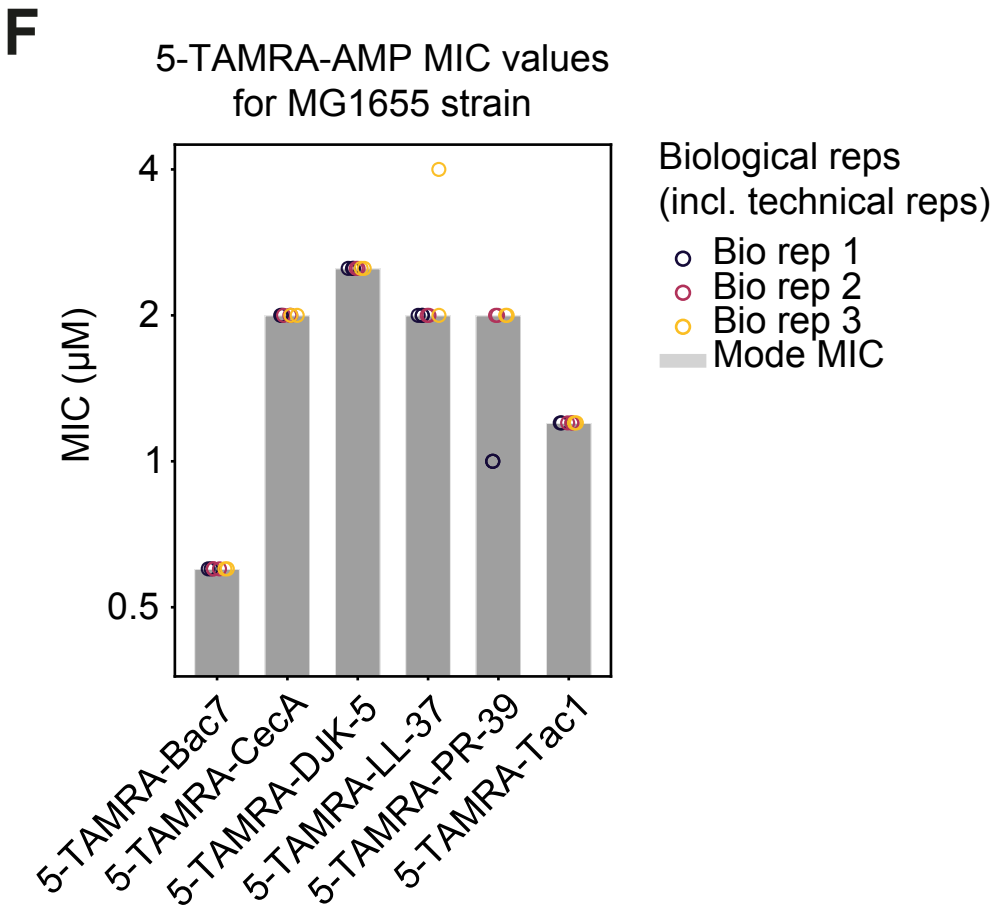


**G**

| AMP derivative | Mode MIC | Unit |
|---|---|---|
| 5-TAMRA-Bac7 | 0.6 | µM |
| 5-TAMRA-CecA | 2 | µM |
| 5-TAMRA-DJK-5 | 2.5 | µM |
| 5-TAMRA-LL-37 | 2 | µM |
| 5-TAMRA-PR-39 | 2 | µM |
| 5-TAMRA-Tac1 | 1.2 | µM |

bioRxiv preprint doi: https://doi.org/10.1101/2025.04.10.648262; this version posted July 24, 2026. The copyright holder for this preprint (which was not certified by peer review) is the author/funder, who has granted bioRxiv a license to display the preprint in perpetuity. It is made available under a CC-BY 4.0 International license.

**Figure S1: Features relevant to the experimental approach. (A)** Data underlying scatterplots shown in Figure 1B for each AMP or PM (excluding peptoids TM1, TM4, TM5) listed in Figure 1A. **(B)** Bar plots showing the mode minimum inhibitory concentration (MIC) across biological replicates for each AMP. peptidomimetic, or antibiotic used in this study, measured in MG1655 *E. coli* cells grown in M9gluCAAT at 37°C for 20 h starting from an inoculum of ~250 cells/mL. Grey bars indicate the mode MIC across three biological replicates (circles), featuring two technical replicates each. **(C)** Table summarizing the mode MIC values and units for all AMPs, peptidomimetics, and antibiotics shown in (B). **(D)** Schematic of the multi-well device used to image cells. The device contains four independent wells in which the growth and division of surface-immobilized E. coli cells can be tracked and AMPs can be added at defined final concentrations. **(E)** Representative fluorescence images of the mScarlet-I signal in CJW7845 cells, illustrating the common enrichment of the periplasmic fluorescent protein at cell poles. Scale bar, 2 µm. **(F)** Same as (A) but for the indicated 5-TAMRA–labeled AMP derivatives used to monitor intracellular accumulation. **(G)** Table summarizing the mode MIC values for the labeled AMP derivatives shown in (F).

bioRxiv preprint doi: https://doi.org/10.1101/2025.04.10.648262; this version posted July 24, 2026. The copyright holder for this preprint (which was not certified by peer review) is the author/funder, who has granted bioRxiv a license to display the preprint in perpetuity. It is made available under a CC-BY 4.0 International license.

## Figure S2

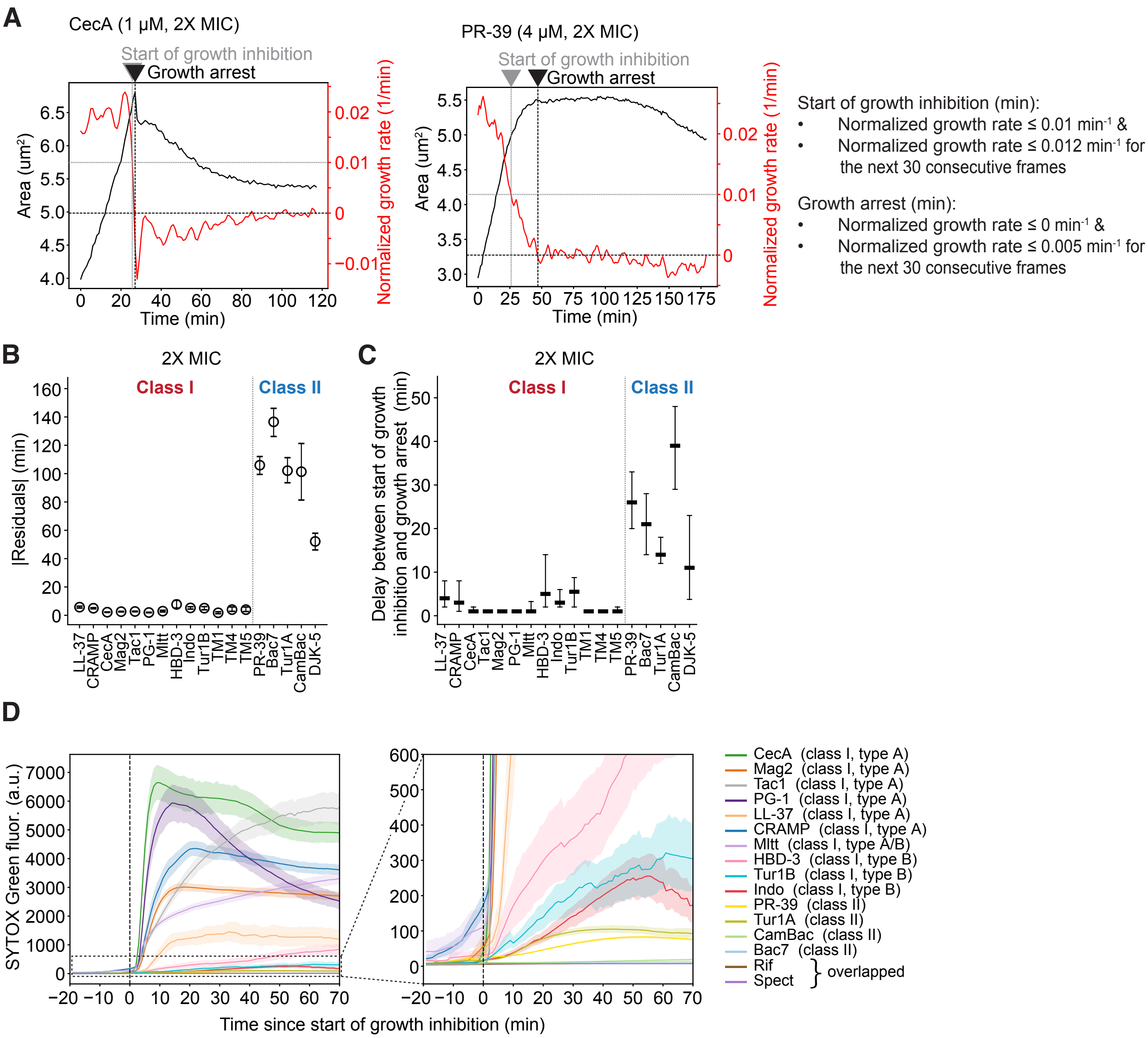


**Figure S2: Determination of growth metrics and SYTOX Green uptake since start of growth inhibition. (A)** Example trajectories of cell area (black) and normalized growth rate (red, as defined in Methods) for a cell treated with CecA (left) or PR-39 (right). Horizontal grey dotted and black dashed lines indicate hard thresholds used for detection of start of growth inhibition or growth arrest, respectively. Vertical grey dotted and black dashed lines indicate the identified times of start of growth inhibition and growth arrest, respectively, using the definitions written on the right. **(B)** Absolute residuals (mean ± 95% confidence interval (CI)) of times of growth arrest since AMP addition vs. times of IM permeabilization since AMP addition from the diagonal ($y = x$), for the indicated AMPs at 2X MIC (same dataset used in Figure 1F). **(C)** Time from the start of growth inhibition to growth arrest (points are medians; error bars represent interquartile range) for the indicated AMPs at 2X MIC (same dataset used in Figure 1F). **(D)** Plot showing SYTOX Green uptake vs time relative to initiation time of growth inhibition for different AMPs at concentrations equivalent to their 2X MICs (see Figure 1A). The left plot shows the full range of measured SYTOX Green values. The right plot shows a zoom-in to visualize the AMPs for which SYTOX Green uptake was lower. The total number of cells was 101, 288, 187, 196, 187, 205, 159, 158, 119, 201, 179, 176, 215, 110, 121, 140 for CecA, Tac1, PG-1, Mag2, LL-37, CRAMP, Mltt, HBD-3, Indo, Tur1B, PR-39, Bac7, Tur1A, CamBac, Rif, and Spect, respectively.

bioRxiv preprint doi: https://doi.org/10.1101/2025.04.10.648262; this version posted July 24, 2026. The copyright holder for this preprint (which was not certified by peer review) is the author/funder, who has granted bioRxiv a license to display the preprint in perpetuity. It is made available under a CC-BY 4.0 International license.

# Figure S3

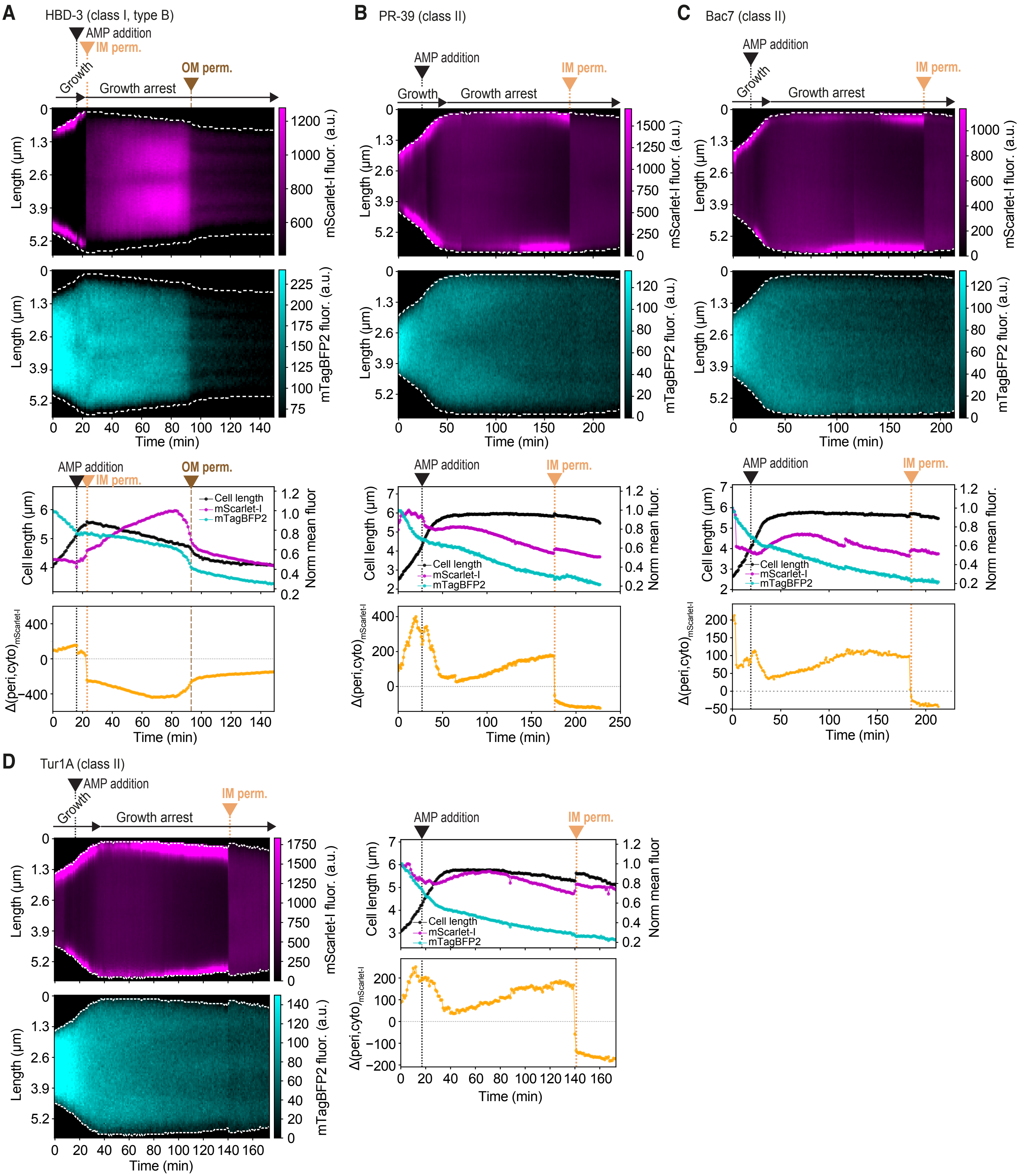

bioRxiv preprint doi: https://doi.org/10.1101/2025.04.10.648262; this version posted July 24, 2026. The copyright holder for this preprint (which was not certified by peer review) is the author/funder, who has granted bioRxiv a license to display the preprint in perpetuity. It is made available under a CC-BY 4.0 International license.

**Figure S3: Example kymographs of mScarlet-I and mTagBFP2 signals for CJW7845 cells upon exposure to HBD-3, PR-39, Bac7, and Tur1A. (A)** *Top*: Example kymographs of mScarlet-I and mTagBFP2 signals for a representative CJW7845 cell exposed with HBD-3 (2X MIC) at the indicated time. The times of IM and OM permeabilizations are shown. *Bottom*: Plots showing the quantification of the cell length and fluorescence signal (top) and the difference Δ(peri,cyto)mScarlet-I between the mScarlet-I signal in the cell periphery (periplasm) relative to the cell interior (cytoplasm) over time for cell shown above. **(B)** Same as (A) but for PR-39 (2X MIC). **(C)** Same as (A) but for Bac7 (2X MIC). **(D)** Same as (A) but for Tur1A (2X MIC).

bioRxiv preprint doi: https://doi.org/10.1101/2025.04.10.648262; this version posted July 24, 2026. The copyright holder for this preprint (which was not certified by peer review) is the author/funder, who has granted bioRxiv a license to display the preprint in perpetuity. It is made available under a CC-BY 4.0 International license.

# Figure S4

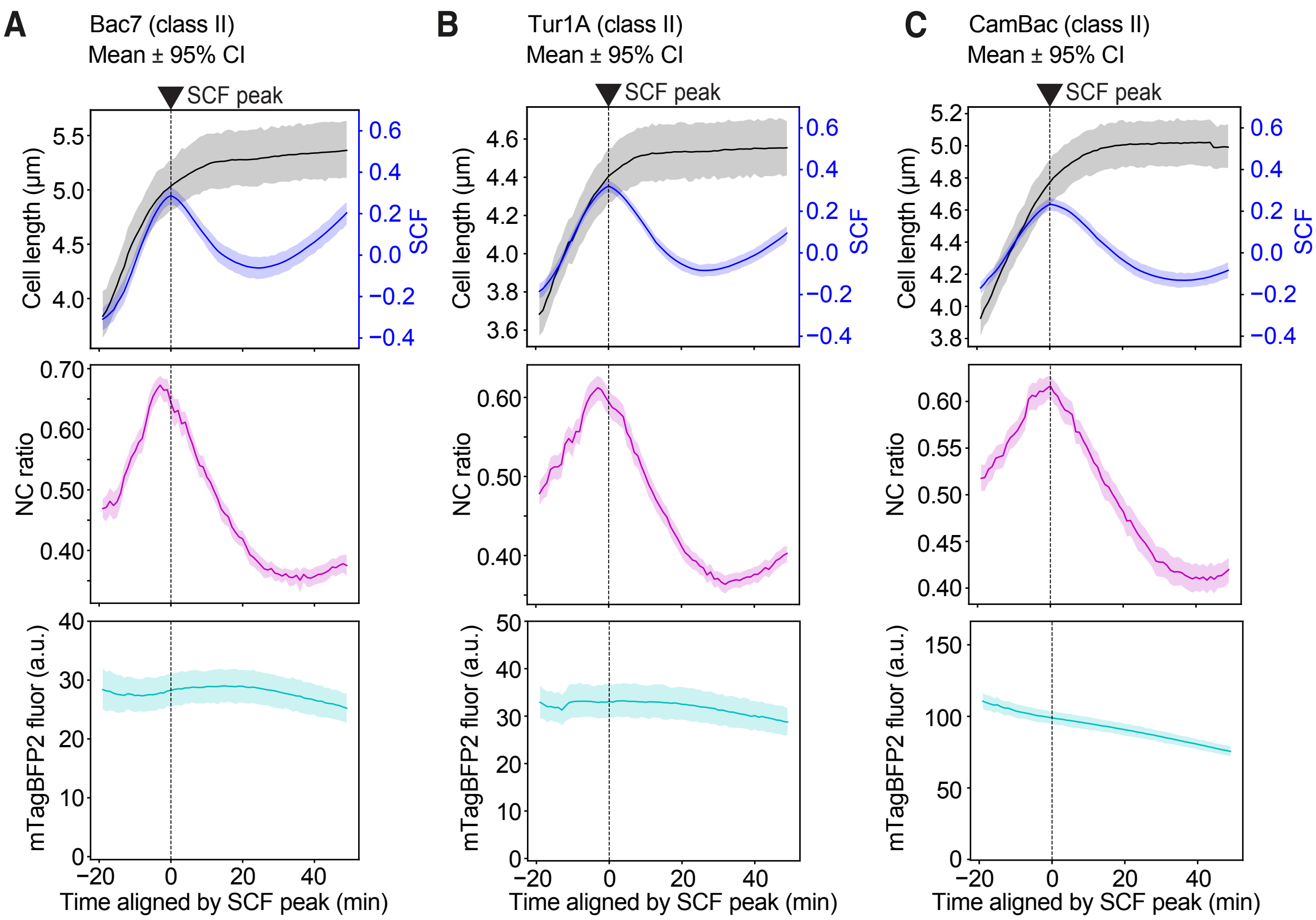


**Figure S4: Quantification of the intracellular DNA and ribosome reorganization upon Bac7, Tur1A, and CamBac treatment. (A)** Plots showing the quantification of the evolution of the mean ± 95% confidence interval (CI) of the cell length, signal correlation factor (SCF), nucleocytoplasmic (NC) ratio, and mTagBFP2 fluorescence (fluor.) across 183 cells exposed to Bac7 at 2X MIC. Times were aligned based on the SCF peak at the single-cell level. The black dashed line indicates the SCF peak (time zero). **(B)** Same as (A) but for Tur1A. The number of cells is 131. **(C)** Same as (A) and (B) but for CamBac. The number of cells is 168.

bioRxiv preprint doi: https://doi.org/10.1101/2025.04.10.648262; this version posted July 24, 2026. The copyright holder for this preprint (which was not certified by peer review) is the author/funder, who has granted bioRxiv a license to display the preprint in perpetuity. It is made available under a CC-BY 4.0 International license.

# Figure S5

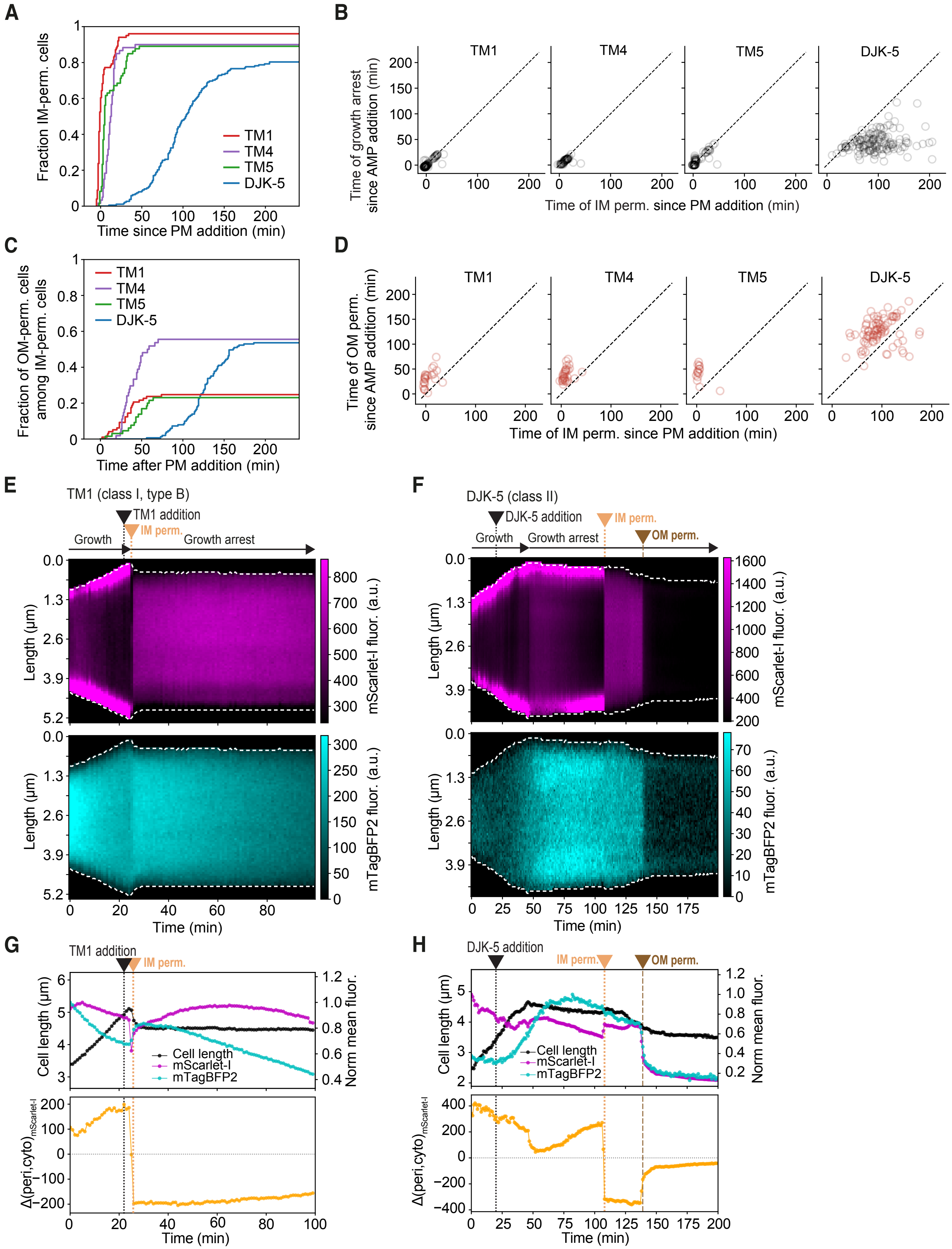

bioRxiv preprint doi: https://doi.org/10.1101/2025.04.10.648262; this version posted July 24, 2026. The copyright holder for this preprint (which was not certified by peer review) is the author/funder, who has granted bioRxiv a license to display the preprint in perpetuity. It is made available under a CC-BY 4.0 International license.

**Figure S5. Phenotype-based classification applies to synthetic AMP mimics.** For all panels, the concentration of peptidomimetics (PM) used was 2X MIC (see Figure 1A). **(A)** Plot showing the cumulative distribution of the fraction of IM-permeabilized cells treated with peptidomimetics (PMs) TM1, TM4, TM5, and DJK-5. The total number of IM-permeabilized cells is 97, 54, 65, and 151 for TM1, TM4, TM5, and DJK-5, respectively. **(B)** Scatterplots showing the time of growth arrest since PM addition vs. that of inner membrane (IM) permeabilization since PM addition for cells treated with TM1, TM4, TM5, and DJK-5. The total number of cells is the same as in (A). Opacity of each data point was set to 15% to aid visualization of the data density. The dashed line indicates $y = x$. **(C)** Plot showing the cumulative distribution of the fraction of OM-permeabilized cells among the IM-permeabilized population during treatment with TM1, TM4, TM5, and DJK-5. The fraction of cells was 24/97 for TM1, 30/54 for TM4, 15/65 for TM5, and 81/151 for DJK-5. **(D)** Scatterplots showing the time of OM permeabilization since PM addition vs. that of IM permeabilization since PM addition for cells treated with TM1, TM4, TM5, and DJK-5, at 2X MIC. The total number of cells is the same as in (C). Opacity of each data point was set to 25% to aid visualization of the data density. The dashed line indicates $y = x$. **(E)** Example kymographs showing the evolution of the mScarlet-I and mTagBFP2 signals for a representative CJW7845 cell exposed with TM1 at. **(F)** Same as (E) but for DJK-5). **(G)** Plots of the cell length, normalized mean fluorescence signals of mScarlet-I and mTagBFP2, and difference Δ(peri,cyto)mScarlet-I between the mScarlet-I fluorescence signal in the cell periphery (periplasm) relative to the cell interior (cytoplasm) over time, for the cell represented in (E), treated with TM1. **(H)** Same as (G) but for the cell in (F) treated with DJK-5.

bioRxiv preprint doi: https://doi.org/10.1101/2025.04.10.648262; this version posted July 24, 2026. The copyright holder for this preprint (which was not certified by peer review) is the author/funder, who has granted bioRxiv a license to display the preprint in perpetuity. It is made available under a CC-BY 4.0 International license.

# Figure S6

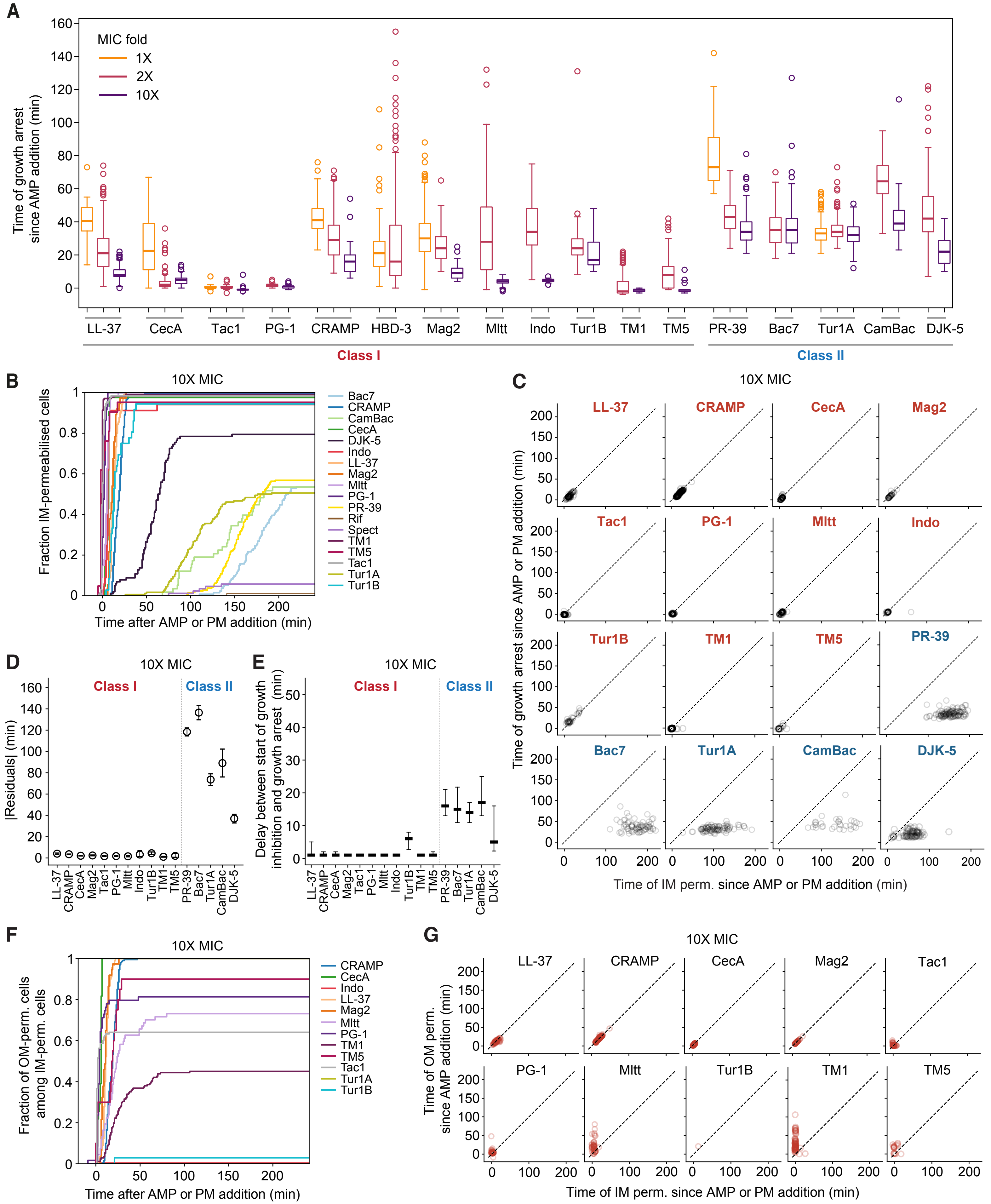

bioRxiv preprint doi: https://doi.org/10.1101/2025.04.10.648262; this version posted July 24, 2026. The copyright holder for this preprint (which was not certified by peer review) is the author/funder, who has granted bioRxiv a license to display the preprint in perpetuity. It is made available under a CC-BY 4.0 International license.

**Figure S6: Quantification of the time delay between AMP addition and growth arrest for all tested AMPs across different peptide concentrations relative to their MICs. (A)** Box plots showing the distribution of times of growth arrest since AMP or peptidomimetic (PM) addition for CJW7845 cells treated at 1X (orange), 2X (pink), and 10X (purple) MIC. AMPs or PMs are separated into class I (left) and class II (right). Boxes show the interquartile range; whiskers extend to 1.5 times the interquartile range; circles indicate outliers. The total number of cells per condition is as follows (1X/2X/10X MIC): CecA (52/100/84), LL-37 (14/167/90), CRAMP (58/194/201), Tac1 (73/274/130), Mag2 (300/181/37), HBD-3 (84/99/–), PG-1 (–/163/59), Mltt (–/156/67), Indo (–/119/34), Tur1B (–/54/36), TM1 (–/101/173), TM4 (–/60/–), TM5 (–/73/21), PR-39 (112/158/213), Bac7 (–/155/134), Tur1A (289/192/180), CamBac (–/98/58), and DJK-5 (–/188/102). **(B)** Cumulative distributions of the fraction of IM-permeabilized CJW7845 cells for all AMPs at 10X MIC, as a function of time since AMP addition. The total number of IM-permeabilized cells is 90, 82, 200, 128, 37, 67, 59, 32, 34, 171, 20, 121, 72, 91, 31, 81, 1, and 5 for LL-37, CecA, CRAMP, Tac1, Mag2, Mltt, PG-1, Indo, Tur1B, TM1, TM5, PR-39, Bac7, Tur1A, CamBac, DJK-5, Rif, and Spect, respectively. **(C)** Scatterplots showing the time of growth arrest since AMP or PM addition vs. that of inner membrane (IM) permeabilization since AMP or PM addition for all indicated AMPs and PMs at 10X MIC. The total number of cells is the same as in (B). Opacity of each data point was set to 15% to aid visualization of the data density. The dashed line indicates $y = x$. **(D)** Absolute residuals of times of growth arrest since AMP addition vs. times of IM permeabilization since AMP or PM addition from the diagonal ($y = x$), for the indicated AMPs and PMs at 10X MIC (same dataset used in (B)). **(E)** Delay between the start of growth inhibition and the growth arrest (points are medians; error bars represent interquartile range) for the indicated AMPs at 10X MIC (same dataset used in (B)). **(F)** Plot showing the cumulative distribution of the fraction of OM-permeabilized cells among the IM-permeabilized population during treatment with the indicated AMPs and PMs at 10X MIC. The total number of OM-permeabilized cells (out of all IM-permeabilized cells) was 90/90 for LL-37, 82/82 for CecA, 200/200 for CRAMP, 82/128 for Tac1, 37/37 for Mag2, 49/67 for Mltt, 48/59 for PG-1, 1/34 for Tur1B, 77/171 for TM1, and 18/20 for TM5. **(G)** Scatterplots showing the time of outer membrane (OM) permeabilization since AMP or PM addition vs. that of inner membrane (IM) permeabilization since AMP or PM addition for cells treated with the indicated AMPs or PMs, at 10X MIC. The total number of cells is the same as in (F). Opacity of each data point was set to 25% to aid visualization of the data density. The dashed line indicates $y = x$.

bioRxiv preprint doi: https://doi.org/10.1101/2025.04.10.648262; this version posted July 24, 2026. The copyright holder for this preprint (which was not certified by peer review) is the author/funder, who has granted bioRxiv a license to display the preprint in perpetuity. It is made available under a CC-BY 4.0 International license.

## Figure S7

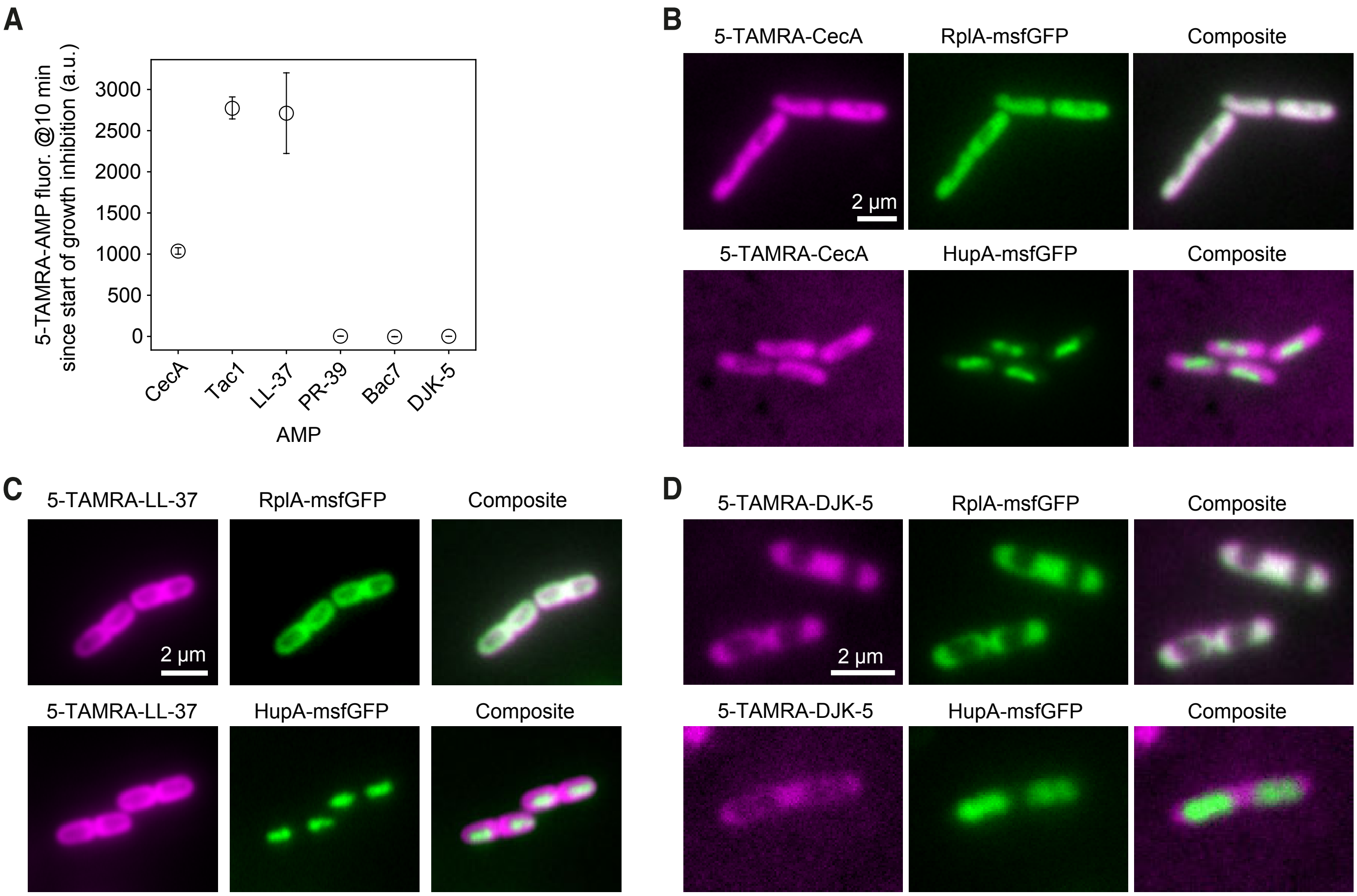


**Figure S7: Intracellular enrichment and spatial localization of fluorescently labeled AMPs. (A)** Intracellular fluorescence intensity (mean± 95 % CI) of 5-TAMRA conjugates to the indicated AMPs 10 min after their addition for MG1655 strain (same data as in Figure 4A) present at 50 nM together with the corresponding unlabeled AMPs (2X MIC) in MG1655 cells. Time is relative to the start time of growth inhibition. The total number of averaged cells is 138, 116, 101, 125, 254, 180, for CecA, Tac1, LL-37, PR-39, Bac7, and DJK-5, respectively. **(B)** Representative images of the indicated fluorescence signal in CJW7020 cells (expressing RplA-msfGFP, top) or CJW7859 (expressing HupA-msfGFP, bottom) exposed to a mixture of CecA (2X MIC) and 5-TAMRA-CecA (50 nM, 5% of the unlabeled version) for 10 min or 20 min, respectively. **(C)** Same as (B) except that cells were exposed to a mixture of LL-37 (2X MIC) and 100 nM (5% of the unlabeled version) of 5-TAMRA-LL-37 for 50 min. **(D)** Same as (B) except that cells were exposed to a mixture of DJK-5 (2X MIC) and 5-TAMRA-DJK-5 (250 nM, 5% of the unlabeled version) for 5 h and 40 min (for CJW7020 cell, top) or 3 h and 10 min (for CJW7859 cells, bottom).

bioRxiv preprint doi: https://doi.org/10.1101/2025.04.10.648262; this version posted July 24, 2026. The copyright holder for this preprint (which was not certified by peer review) is the author/funder, who has granted bioRxiv a license to display the preprint in perpetuity. It is made available under a CC-BY 4.0 International license.

## Figure S8

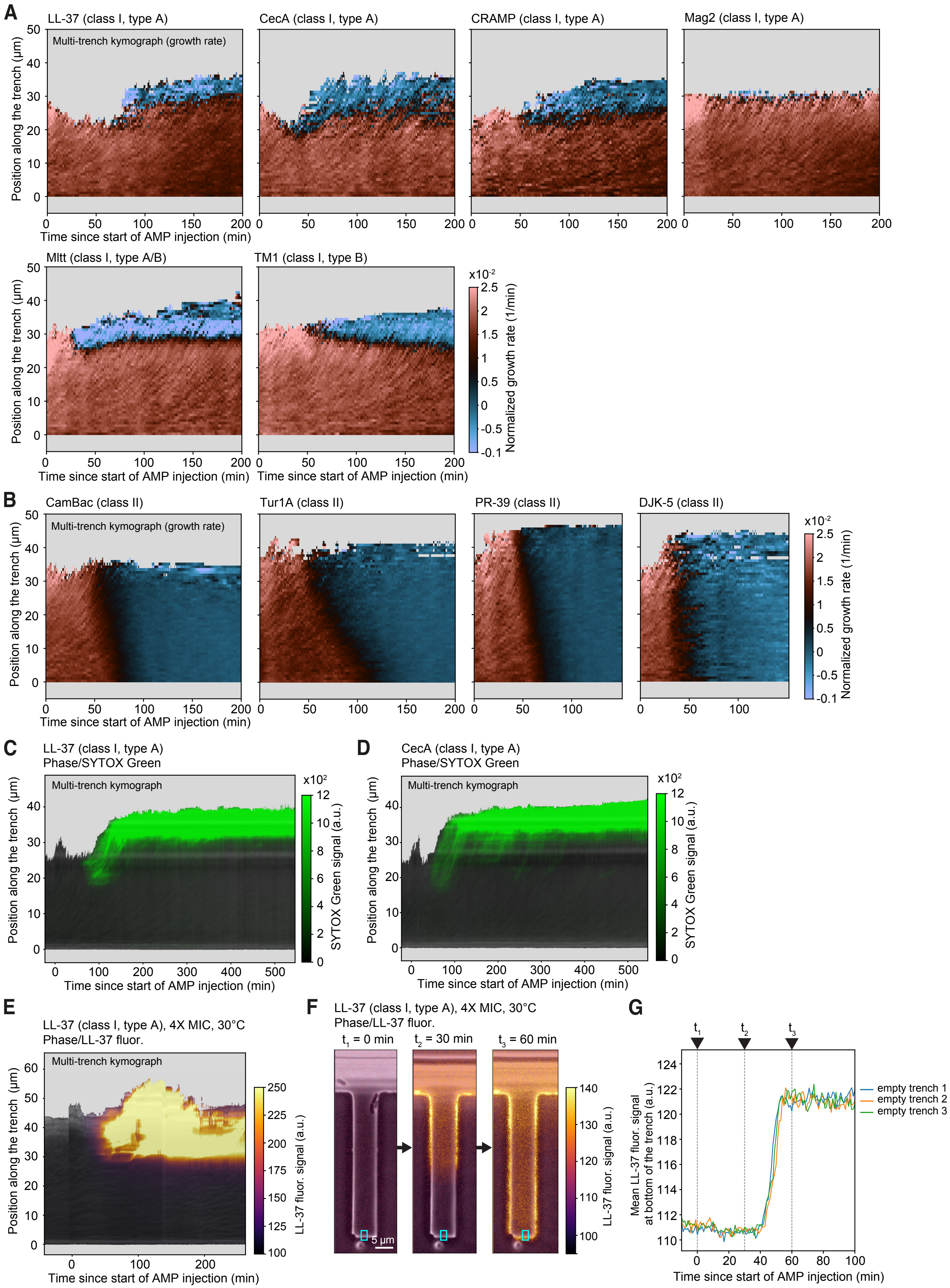

bioRxiv preprint doi: https://doi.org/10.1101/2025.04.10.648262; this version posted July 24, 2026. The copyright holder for this preprint (which was not certified by peer review) is the author/funder, who has granted bioRxiv a license to display the preprint in perpetuity. It is made available under a CC-BY 4.0 International license.

**Figure S8: Ensemble kymographs of growth rate, SYTOX Green, and LL-37 fluorescence in wide trenches. (A)** Ensemble kymographs of the normalized growth rate distribution along wide trenches over time, computed by averaging across multiple trenches for CJW7753 cells treated with the indicated class I AMPs or PMs at 2X MIC: The number of trenches is 18, 24, 12, 23, 18, and 17, and the number of cells is 5012, 5081, 2590, 6707, 6317, and 6266 for LL-37, CecA, CRAMP, Mag2, Mltt, and TM1, respectively. **(B)** Same as (A) but for CJW7753 cells treated with the indicated class II peptides at 2X MIC. The number of trenches is 32, 28, 27, and 28, and the number of cells is 4189, 4210, 4312, and 2989 for CamBac, Tur1A, PR-39, and DJK-5, respectively. **(C)** Multi-trench kymograph showing the ensemble average of phase-contrast (grey) and SYTOX Green fluorescence (green) along wide trenches over time for CJW7845 cells treated with LL-37 (class I type A) at 2X MIC. The kymograph was computed by averaging across 24 trenches. SYTOX Green signal represents cells with permeabilized membranes. **(D)** Same as (C) but for CJW7845 cells treated with CecA (class I type A), computed from 24 trenches. **(E)** Multi-trench kymograph showing phase-contrast and LL-37 fluorescence signal along wide trenches over time for CJW7859 cells (expressing HupA-msfGFP) treated with LL-37 (4X MIC) at 30°C, averaged across 18 trenches. The fluorescence reports on LL-37 absorption by cells. **(F)** Representative fluorescence images of a single empty trench at three selected timepoints ($t_0$, $t_1$, $t_2$) illustrating how LL-37 can diffuse all the way to the bottom of the empty trench within 60 min from the start of AMP injection. Cyan colored box represents the ROI where LL-37 signal intensity was computed over time and shown in (G). **(G)** Plot showing LL-37 fluorescence intensity as a function of time since AMP injection, measured at the bottom of three empty trenches by averaging pixel intensities within a defined ROI (as illustrated in (F)).

bioRxiv preprint doi: https://doi.org/10.1101/2025.04.10.648262; this version posted July 24, 2026. The copyright holder for this preprint (which was not certified by peer review) is the author/funder, who has granted bioRxiv a license to display the preprint in perpetuity. It is made available under a CC-BY 4.0 International license.

## Figure S9

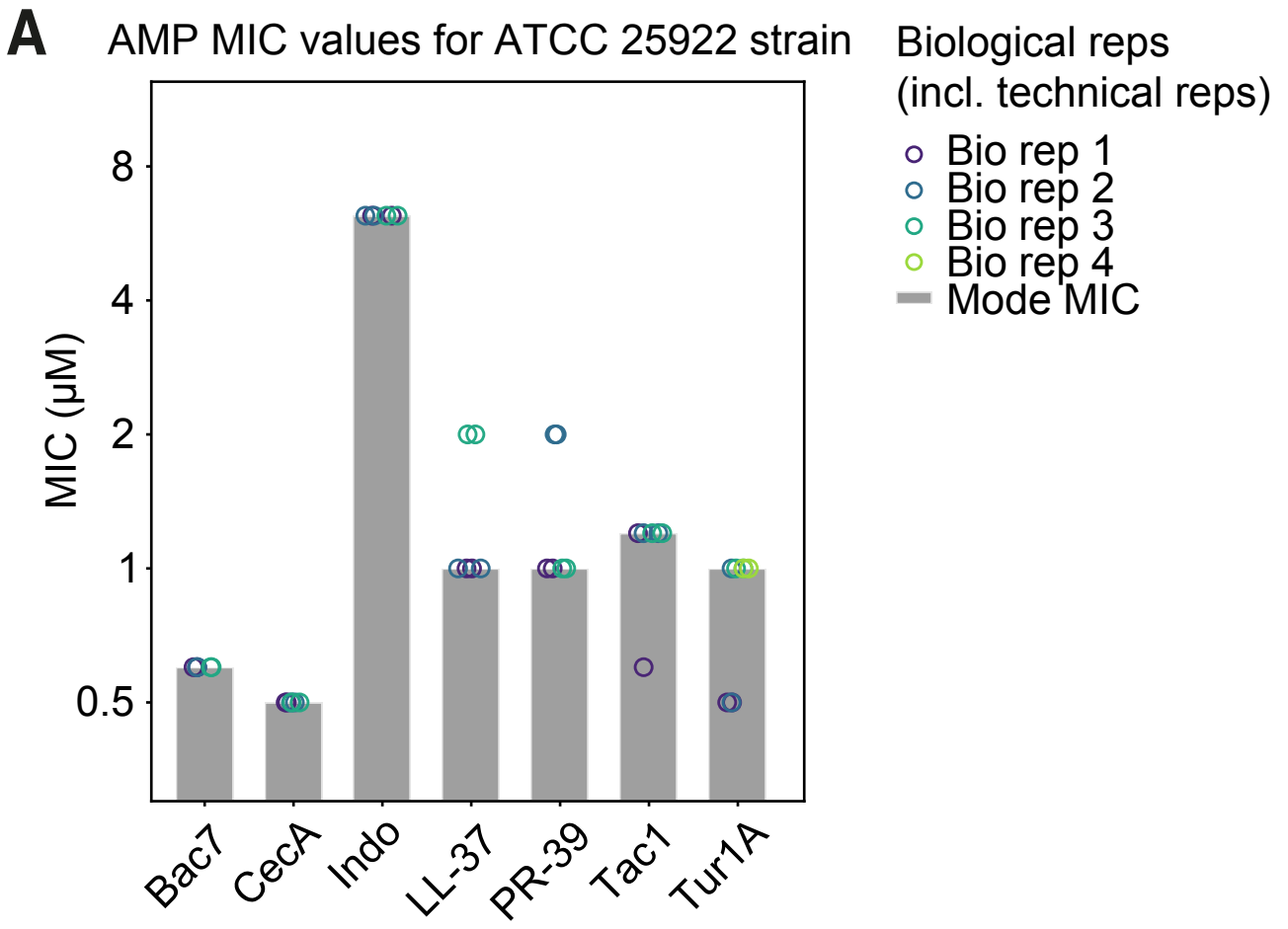


B

| AMP | Mode MIC | Unit |
|---|---|---|
| Bac7 | 0.6 | µM |
| CecA | 0.5 | µM |
| Indo | 6.2 | µM |
| LL-37 | 1 | µM |
| PR-39 | 1 | µM |
| Tac1 | 1.2 | µM |
| Tur1A | 1 | µM |

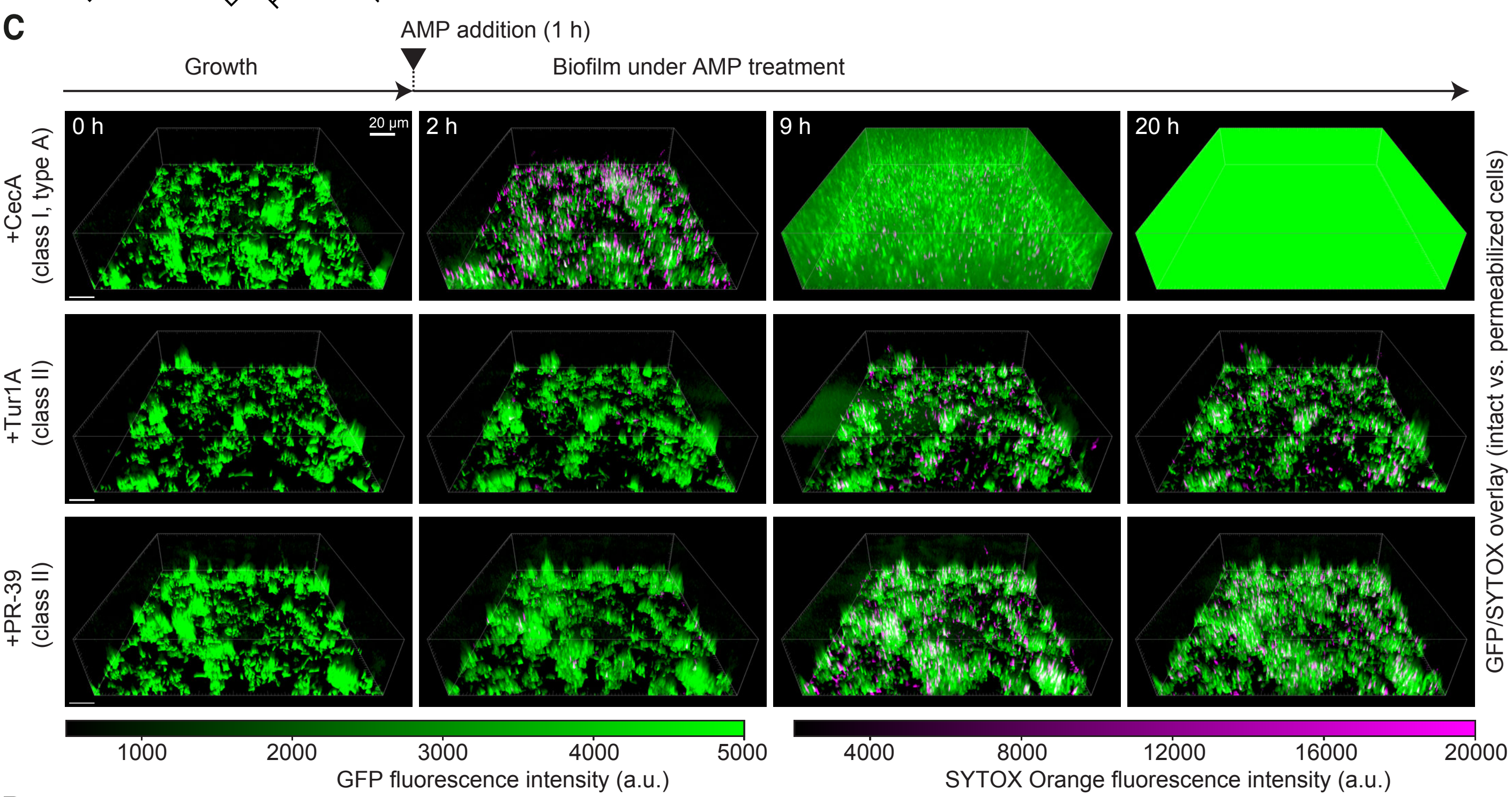


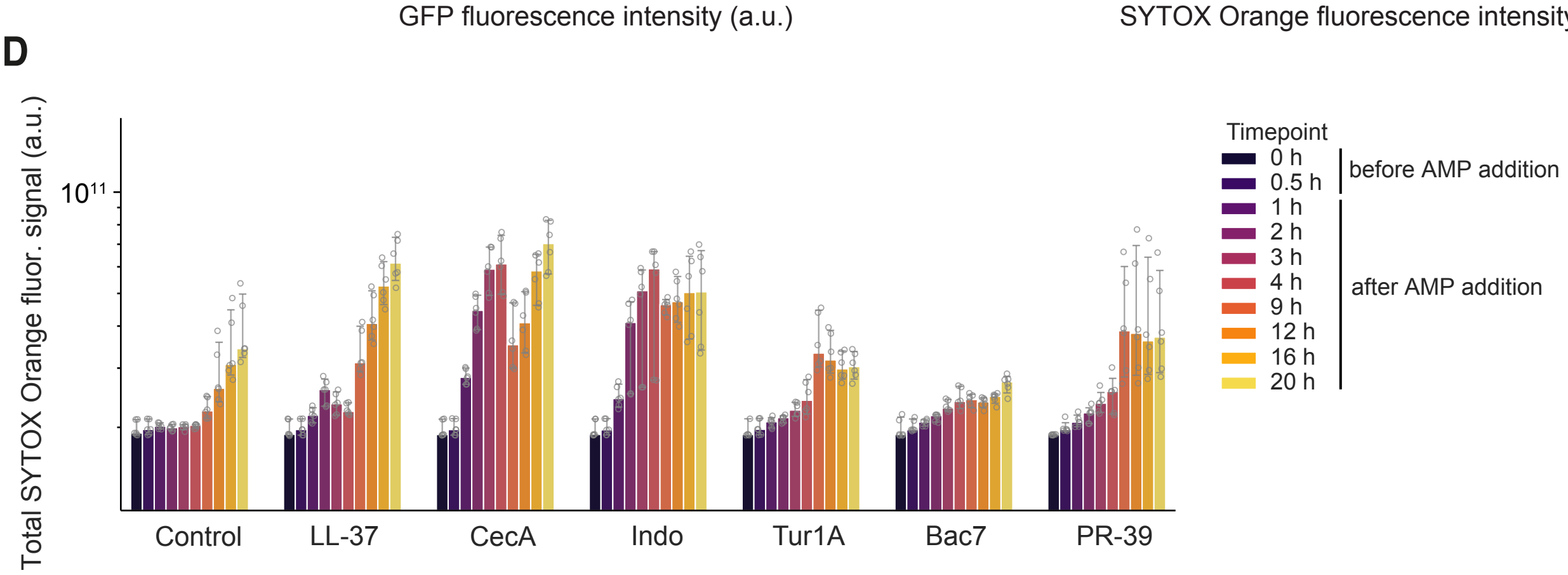


**Figure S9: Biofilm growth under AMP treatment. (A)** Bar plot showing the mode MIC across biological replicates for the indicated AMPs measured against E. coli ATCC 25922 in M9gluCAAT at 37°C for 20 h. Grey bars indicate the mode MIC across four biological replicates (circles). **(B)** Table summarizing the mode MIC values (in µM) for each AMP shown in (A). **(C)** Selected timepoints of biofilms treated with (or without) AMPs, showing overlaid GFP (cells) and SYTOX Orange (permeabilized cells) signals. AMPs were added at 2X MIC ~1 h after the start of image acquisition. **(D)** Bar plot showing the quantification of the total SYTOX Orange signal within the imaged 3D volume over time for the indicated conditions. Fluorescence signals in (C) were rendered and exported from Imaris with gamma set to 2 for display only.

bioRxiv preprint doi: https://doi.org/10.1101/2025.04.10.648262; this version posted July 24, 2026. The copyright holder for this preprint (which was not certified by peer review) is the author/funder, who has granted bioRxiv a license to display the preprint in perpetuity. It is made available under a CC-BY 4.0 International license.

# Figure S10

**A**

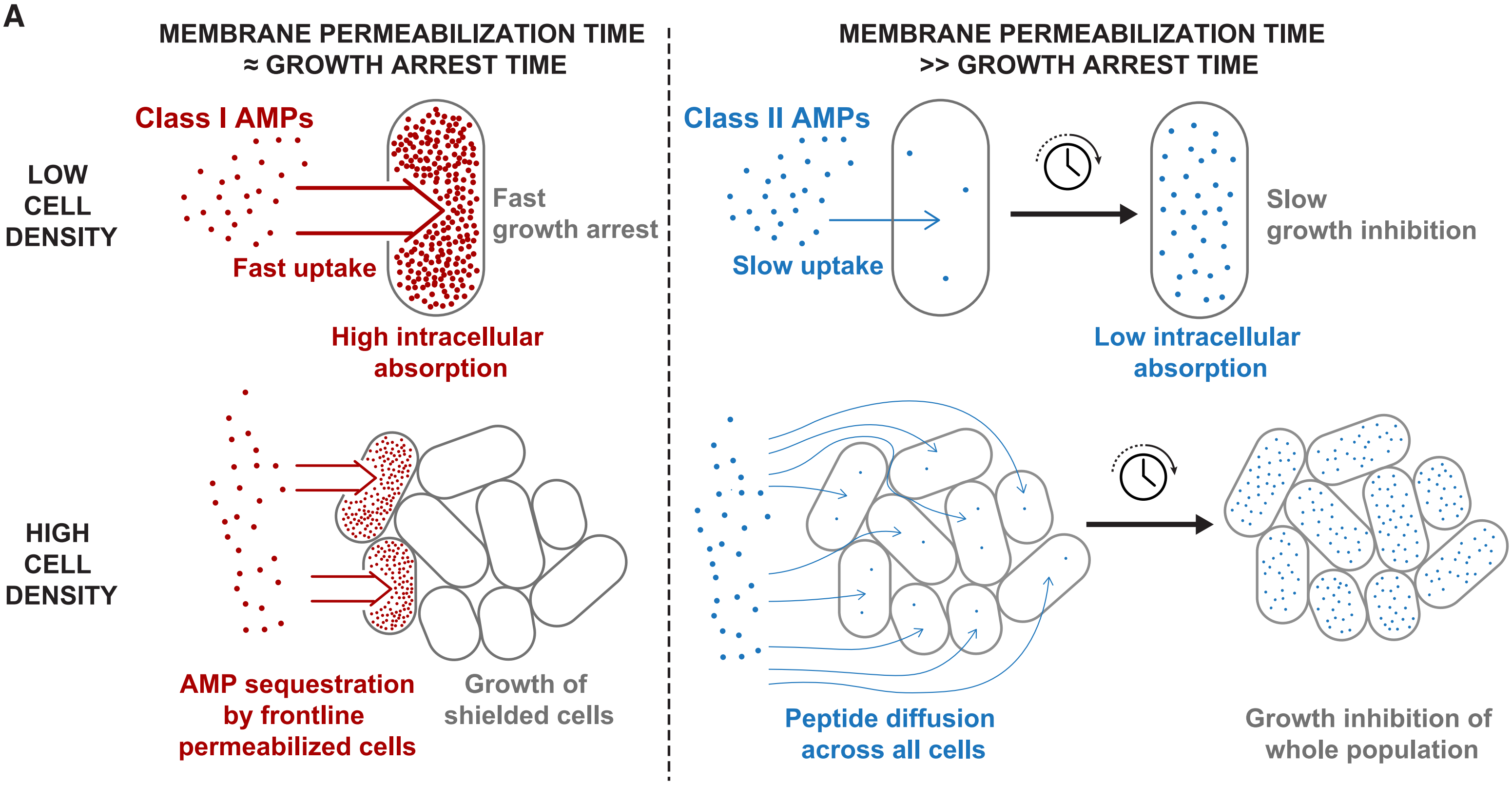


**B**

| Name (abbreviation) | Sequence | Class | Type |
|---|---|---|---|
| LL-37 | LLGDFFRKSKEKIGKEFKRIVQRIKDFLRNLVPRTES | I | A |
| Cecropin A (CecA) | KWKLFKKIEKVGQNIRDGIIKAGPAVAVVGQATQIAK-$NH_2$ | I | A |
| Magainin-2 (Mag2) | GIGKFLHSAKKFGKAFVGEIMNS | I | A |
| CRAMP | GLLRKGGEKIGEKLKKIGQKIKNFFQKLVPQPEQ | I | A |
| Tachyplesin-1 (Tac1) | KWCFRVCYRGICYRRCR-$NH_2$ | I | A |
| Protegrin-1 (PG-1) | RGGRLCYCRRRFCVCVGR-$NH_2$ | I | A |
| Melittin (Mltt) | GIGAVLKVLTTGLPALISWIKRKRQQ-$NH_2$ | I | A/B |
| Human beta-defensin 3 (HBD-3) | GIINTLQKYYCRVRGGRCAVLSCLPKEEQIGKCSTRGRKCCRRKK | I | B |
| Indolicidin (Indo) | ILPWKWPWWPWRR-$NH_2$ | I | B |
| Tur1B | RRIPFWPPNWPGPWLPPWSPPDFRIPRILRKR | I | B |
| TM1 | H-(NLys-Nspe-Nspe)$_4$-$NH_2$ | I | B |
| TM4 | H-(NLys-Nspe(p-Br-Nspe(p-Br))$_2$-$NH_2$ | I | B |
| TM5 | H-Ntridec-NLys-Nspe-Nspe-NLys-$NH_2$ | I | B |
| PR-39 | RRRPRPPYLPRPRPPPFFPPRLPPRIPPGFPPRFPPRFP-$NH_2$ | II | B |
| Bactenecin-7 (Bac7) | RRIRPRPPRLPRPRPRPLPFPRPGPRPIPRPLPFP | II | B |
| Tur1A | RRIRFRPPYLPRPGRRPRFPPPFPIPRIPRIP | II | B |
| Camel Bactenecin (CamBac) | RSIRRPRLPRPRVPRPYIPPRIPRPVLPPPRFPIPRFPRGR | II | B |
| DJK-5 | VQWRAIRVRVIR-$NH_2$ | II | B |

bioRxiv preprint doi: https://doi.org/10.1101/2025.04.10.648262; this version posted July 24, 2026. The copyright holder for this preprint (which was not certified by peer review) is the author/funder, who has granted bioRxiv a license to display the preprint in perpetuity. It is made available under a CC-BY 4.0 International license.

**Figure S10. Summary of the functional trade-offs and AMP classification. (A)** Class I AMPs (red) couple fast membrane permeabilization with rapid growth arrest. At low cell density, fast peptide uptake leads to high intracellular accumulation. At high cell density, frontline permeabilized cells rapidly sequester the bulk of available peptide, shielding the cells behind them from exposure. Class II AMPs (blue) permeabilize membranes more slowly than they inhibit growth, resulting in slow, gradual intracellular accumulation and low absorption per cell. At high cell density, this slow uptake allows peptides to diffuse across the population before any individual cell sequesters a significant fraction, ultimately inhibiting growth of the whole population. **(B)** Sequences and phenotypic classification of the AMPs and PMs used in this study. Class I AMPs and PMs cause growth arrest predominantly associated with inner membrane (IM) permeabilization, whereas class II compounds inhibit growth without substantial IM permeabilization. Within class I, type A AMPs predominantly permeabilize the outer membrane (OM) within 10 min of the IM, whereas type B AMPs and PMs permeabilize the IM well before (>10 min) the OM. All class II peptides are found to be type B. Melittin (Mltt) displays intermediate behavior and is classified as type A/B.
